# Seroprevalence of Anti-Cytomegalovirus Antibodies in Pregnant Women from South-West Romania

**DOI:** 10.3390/microorganisms12020268

**Published:** 2024-01-26

**Authors:** Cristiana Luiza Radoi, Ovidiu Zlatian, Maria Balasoiu, Tiberiu-Liviu Dragomir, Madalina Ioana Sorop, Iulia Cristina Bagiu, Estera Boeriu, Monica Susan, Bogdan Sorop, Licinia Andrada Oprisoni, Dominic Gabriel Iliescu

**Affiliations:** 1 Doctoral School, University of Medicine and Pharmacy of Craiova, 200349 Craiova, Romania; luizacristianaradoi@gmail.com; 2 Medical Laboratory, County Clinical Emergency Hospital of Craiova, 200349 Craiova, Romania; ovidiu.zlatian@umfcv.ro (O.Z.); maria.balasoiu@umfcv.ro (M.B.); 3 Microbiology Department, University of Medicine and Pharmacy of Craiova, 200349 Craiova, Romania; 4 Department of Internal Medicine I, “Victor Babes” University of Medicine and Pharmacy, Eftimie Murgu Square 2, 300041 Timisoara, Romania; dragomir.tiberiu@umft.ro; 5 Doctoral School, “Victor Babes” University of Medicine and Pharmacy, 300041 Timisoara, Romania; pop_madalina_91@yahoo.ro; 6 Department of Microbiology, “Victor Babes” University of Medicine and Pharmacy, Eftimie Murgu Square 2, 300041 Timisoara, Romania; bagiu.iulia@umft.ro; 7 Multidisciplinary Research Center on Antimicrobial Resistance (MULTI-REZ), Microbiology Department, “Victor Babes” University of Medicine and Pharmacy, Eftimie Murgu Square 2, 300041 Timisoara, Romania; 8 Department of Pediatrics, “Victor Babes” University of Medicine and Pharmacy, Eftimie Murgu Square 2, 300041 Timisoara, Romania; 9 Department of Internal Medicine I, Centre for Preventive Medicine,”Victor Babes” University of Medicine and Pharmacy, Eftimie Murgu Sq. No. 2, 300041 Timisoara, Romania; susan.monica@umft.ro; 10 Department of Obstetrics and Gynecology, “Victor Babes” University of Medicine and Pharmacy, Eftimie Murgu Square, No. 2, 300041 Timisoara, Romania; bogdan.sorop@umft.ro; 11 Department of Pediatrics, Discipline of Pediatric Oncology and Hematology, “Victor Babes” University of Medicine and Pharmacy, Eftimie Murgu Square, No. 2, 300041 Timisoara, Romania; oprisoni.licinia@umft.ro; 12 Obstetrics and Gynecology Department, County Clinical Emergency Hospital of Craiova, 200349 Craiova, Romania; dominic.iliescu@gmail.com; 13 Obstetrics and Gynecology Department, University of Medicine and Pharmacy of Craiova, 200349 Craiova, Romania

**Keywords:** cytomegalovirus, seroprevalence studies, congenital infections, pregnant women, antibodies

## Abstract

Cytomegalovirus (CMV), in addition to other agents, is part of the TORCH complex (Toxoplasma gondii, Rubella virus, Cytomegalovirus, Herpes simplex viruses, and other agents). CMV infection is the most frequent cause of congenital malformations. This study aimed to establish the variation of prevalence of anti-CMV antibodies in pregnant women from the South-West region of Romania, according to demographic factors, such as age and area of residence, in two separate time periods (2013–2016 and 2019–2022). We collected from the hospital records the age, place of residence, and anti-CMV antibody test results using immune electrochemiluminescence and chemiluminescence. This study found that the seroprevalence of anti-CMV IgM antibodies increased slightly from 2013–2016 to 2019–2022, from 1.92% to 2.26%, and for IgG antibodies from 93.68% to 94.96%. In both groups was observed a descending trend of anti-CMV IgM seroprevalence with an increase in age, showing a decrease in seroprevalence from 3.57% to 1.09% in pregnant women from rural areas in the 31–35 years age group, while in urban areas, we observed a decrease in seroprevalence from 11.11% to 3.06% in the <20 years age group. The IgG seroprevalence showed an increase both in rural areas (from 93.97% to 95.52%) and urban areas (from 93.52% to 94.27%). In both groups, seroprevalence was higher in rural areas compared to urban regions. These results show a high rate of immunization against CMV in pregnant women in South-West Romania, which led to a low risk of acquiring the primary infection during pregnancy. However, the increase in the rate of primary CMV infections in pregnancy suggests the need for prioritizing screening programs and improving the existing protocols to enhance maternal and child healthcare.

## 1. Introduction

Cytomegalovirus (CMV), also known as human herpesvirus 5, is a common herpesvirus that often causes asymptomatic infections but can lead to severe complications in immunocompromised patients and congenital diseases [1]. Congenital CMV infections are characterized by a long-term balance between the host and virus, resulting in latent infections. The risk of mother-to-child transmission is 30–35% in primary maternal infections, but only 1–2% in non-primary or recurrent infections [2,3,4,5]. In immunocompetent individuals, CMV-related lesions are minimal or non-existent, while in immunocompromised hosts, they can be extensive and affect multiple organs [5].

CMV has a global distribution and can be a significant concern in organ transplantation, cardiac interventions, and blood transfusions, especially for immunodeficient individuals [6,7], requiring serological screening [8]. The virus’s prevalence is widespread, as 24.6–81.0% of healthy individuals show positive anti-CMV IgG antibodies, and congenital CMV infection has a global prevalence of 0.5–5% from live births [3].

Pregnant women without CMV immunization face a 1–4% risk of primary infection, with a 30–40% chance of neonatal CMV infection. Those with past CMV infection have a 1–14% risk of non-primary infection [9]. In Romania, the anti-CMV IgM seroprevalence rates vary, with significant differences observed across regions [10,11,12].

In Europe, the seroprevalence of anti-CMV IgG antibodies varies from 45.6% to 95.7% [13,14,15,16]. In pregnant women from Western Romania, the seroprevalence exhibited a variation, ranging from 94.6% during 2008–2010 to 91.8% in the period of 2015–2018 [12]. A 2021 study documented these findings with comparable results [11]. We could not find in the literature recent data about anti-CMV seroprevalence from South-West Romania.

CMV is the most common infection transmitted in utero from mother to child, at a rate of 1–4% [17]. CMV transmission can occur via multiple routes, including in utero, during childbirth and breastfeeding, airborne transmission, and through contact with infected body fluids. The most common in utero transmission occurs from mothers with primary infection, leading to a range of possible fetal complications [18] regardless of the gestational age but dependent on maternal immune resistance [17,19,20]. Approximately 10% of in utero infected children show signs of infection at birth.

While there are some extensive studies on CMV infection among pregnant women in Western Romania, South-West Romania lacks similar large-scale research. This investigation stands as the first comprehensive study on maternal seroprevalence of anti-CMV IgM antibodies and immunization status in pregnant women from South-West Romania, thereby addressing a notable gap in existing data. Additionally, it investigates risk factors, such as age and area of residence, associated with these infections, analyzing patient data from two distinct periods (2013–2016 and 2019–2022) across a decade, to understand the dynamic nature of these infections in the region.

## 2. Materials and Methods

### 2.1. Study Design

This was a retrospective, observational, and cross-sectional study, conducted on two cohorts of pregnant women who consecutively presented at the Emergency County Clinical Hospital of Craiova, Romania. National health data show that in South-West Romania between 2019 and 2022 were registered 37,001 pregnant women [21]. By calculating the sample size using the lowest reported CMV seroprevalence in Romania of 91.8% in the year 2020 [12], it results in a minimum sample size of 116 pregnant women with a confidence level of 95% that the real value is within ±5% of the measured value. Because we used 653 pregnant women in the first period from South-West Romania, we have a margin of error of ±2.09%. In the second period, the sample size of 3221 pregnant women gives a margin of error of ±0.91%.

The study participants were selected from two distinct time periods, 2013–2016 (Group 1) and 2019–2022 (Group 2), to evaluate temporal shifts in anti-CMV antibody seroprevalence. As we aimed to observe the dynamic change in the seroprevalence of anti-CMV antibodies between two distinct timeframes, each spanning four years, to ensure meaningful results, we chose to have a substantial gap between the chosen periods relative to their duration, as seen in other Romanian studies [12]. Group 1 included 653 participants, while Group 2 consisted of 3221 participants.

Participants were pregnant Romanian women from various counties in the South-West region of Romania (Dolj, Gorj, Olt, Mehedinti, Vâlcea). These women were undergoing routine hospital-based screening for antibodies against TORCH agents.

The study received approval from the Committee of Ethics and Academic and Scientific Deontology in Craiova, Romania (Decision No. 84/16 September 2020) and conformed to the ethical standards and legal mandates of Romania for such research.

### 2.2. Data Collection

For each study participant we collected the following data: demographic and clinical data, including age and area of residence, and serology results for anti-CMV IgM and IgG antibodies.

### 2.3. Serological Testing of Anti-CMV Antibodies

The detection of anti-CMV IgM and IgG antibodies was performed using distinct methodologies tailored to each group of pregnant women. In Group 1, we employed an immune electrochemical luminescence assay (Cobas E601, Roche Diagnostics, Basel, Switzerland) for serological analysis. This assay utilizes a state-of-the-art platform known for its precision and sensitivity in detecting antibodies. In Group 2, our approach shifted to an immune chemiluminescence assay conducted on the Architect i1000 analyzer (Abbott Laboratories, Abbott Park, IL, USA). This analyzer is recognized for its high-throughput capabilities, allowing for efficient testing of a larger cohort of participants. For both groups, all necessary reagents and test kits were procured directly from the respective manufacturing companies, ensuring the reliability and consistency of the assays. The tests were verified each day by internal controls and the calibration was performed periodically using reagents from the producer.

The hands-on methodology consisted simply of centrifugation for 10 min at 2500 RPMs of the sera collected in tubes with clot accelerant, transferring 0.25 mL of the supernatant in sterile tubes provided by the manufacturer, and inserting these tubes into the analyzer. Briefly, the assay [22] followed a competition principle, beginning with pre-treatment of the sample using a reducing agent. Biotinylated antibodies and ruthenium-labeled antibodies specific to CMV antigens are added, along with streptavidin-coated microparticles. The entire complex binds to a solid phase through biotin–streptavidin interaction, and microparticles are magnetically captured on an electrode’s surface in a measuring cell. Application of voltage induces chemiluminescence, which is measured by a photomultiplier.

The interpretation of the cutoff values as positive or negative for anti-CMV antibodies followed strict adherence to the manufacturer’s guidelines. These guidelines provided clear and standardized criteria for determining the presence or absence of antibodies in the participants’ serum samples, maintaining the integrity and consistency of our seroprevalence data across both study groups [23].

### 2.4. Statistical Analysis

Patient data were meticulously collected using the HIPOCRATE software (version H3 Concept) system implemented within the hospital. Subsequently, these data were transferred to STATA 17 (StataCorp Ltd., College Station, TX, USA) for comprehensive statistical analysis. In presenting the data, ratios were expressed as percentages, and the mean age was detailed as a mean value accompanied by the standard deviation. To evaluate differences in mean age between the groups, Student’s *t*-test was used. For analyzing variations in participant distribution across different age groups, an analysis of variance (ANOVA) test, supplemented with Bonferroni correction for multiple comparisons, was used. Additionally, the non-parametric Cochran–Armitage test was applied to identify trends in seroprevalence relative to age groups.

## 3. Results

### 3.1. Anti-CMV IgM Antibodies in Pregnant Women

Demographic characteristics. Group 1 included 572 pregnant women tested between 2013 and 2016, and Group 2 included 2832 participants tested between 2019 and 2022. In Group 1, a majority (65.74%) resided in urban areas (Table 1), whereas in Group 2, the representation of urban dwellers (42.87%) was slightly less than that of rural residents (57.13%) (*p* < 0.001). However, the mean age of women in both groups did not demonstrate a statistically significant difference (*p* = 0.595).

**Figure 1:**
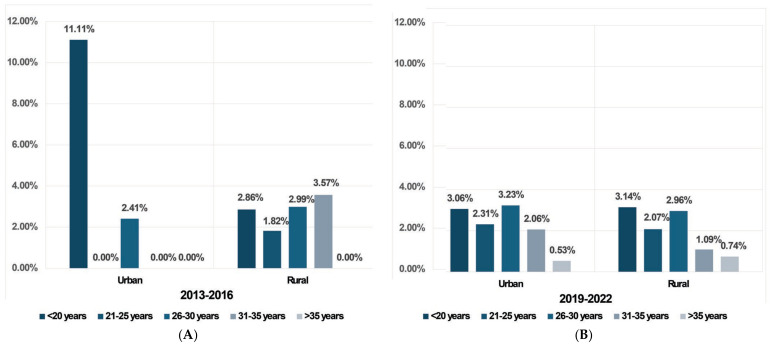
Seroprevalence of anti-CMV IgM antibodies in the two groups according to demographic factors: (**A**) Group 1 (2013–2016); (**B**) Group 2 (2019–2022).

Seroprevalence results. The total seroprevalence of anti-CMV IgM antibodies increased from 1.92% in 2013–2016 to 2.26% in 2019–2022. In Group 1, rural areas showed a higher prevalence (2.55%) compared to urban areas (1.60%), whereas in Group 2, the urban prevalence (2.31%) surpassed that of rural areas (2.22%). This indicates a minor decrease in seroprevalence in rural areas and an increase in urban areas over time (Table 2, Figure 1).

The incidence of acute CMV infection showed variation with age. In both groups, the highest prevalence was noted in the <20 years (5.66% in Group 1 and 3.13% in Group 2).

Analyzing the seroprevalence by age and area of residence we observed that in Group 1 the highest seroprevalence in rural areas was found in the 31–35 years age group (3.57%). In urban regions, the peak seroprevalence was 11.11% in pregnant women aged below 20 years (Table 2 and Figure 1). The results showed a decrease in seroprevalence from 3.57% to 1.09% in pregnant women from rural areas in the 31–35 years age group, while in urban areas, we observed a decrease in seroprevalence from 11.11% to 3.06% in <20 years age group (Table 2).

In Group 2, in rural areas, the highest seroprevalence was 3.14% (<20 years age group), while in urban areas the highest seroprevalence was in the 26–30 years age group (3.23%), as detailed in Table 2 and Figure 1.

In both groups, a descending trend of seroprevalence with an increase in age was observed in women older than 25 years (Table 2). An upward trend from Group 1 to Group 2 was observed in older age groups, though none of these changes reached statistical significance (*p* > 0.05).

### 3.2. Anti-CMV IgG Antibodies in Pregnant Women

Demographic characteristics. We tested 585 pregnant women from 2013 to 2016 (34.02% from rural environment) and 2399 pregnant women between 2019 and 2022 (54.90% rural). The residential distribution between the two groups was statistically different (*p* < 0.001) (Table 3). No significant difference was noted between the mean age between the two groups.

Seroprevalence results. We observed an increase in the total seroprevalence of anti-CMV IgG antibodies from 93.68% between 2013 and 2016 to 94.96% between 2019 and 2022. The increase was observed both in rural areas (from 93.97% to 95.52%) and urban areas (from 93.52% to 94.27%). In both groups, seroprevalence was slightly higher in rural areas compared to urban regions.

**Figure 2:**
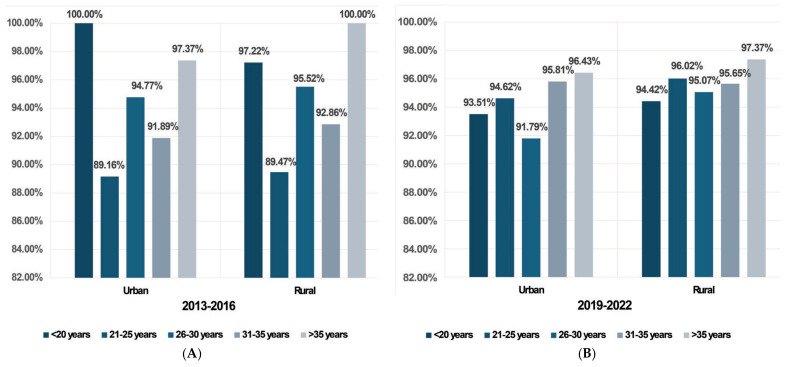
Seroprevalence of anti-CMV IgG antibodies in the two groups according to demographic factors: (**A**) Group 1 (2013–2016); (**B**) Group 2 (2019–2022).

When stratified by age, the highest seroprevalence of anti-CMV IgG antibodies during 2013–2016 was in the under 20 years group (98.18%). Conversely, between 2019 and 2022, the highest seroprevalence was observed in the over 35 years age group (273/282, 96.81%). Across all age groups, the only statistically significant increase in seroprevalence was noted in the 21–25 years age group, from 89.29% in Group 1 to 95.56% in Group 2 (*p* = 0.004), which maintained when stratified by area of residence (Table 4 and Figure 2).

### 3.3. Anti-CMV IgM Antibodies in Immunized Pregnant Women

In Group 1, we observed an increased anti-CMV IgM seroprevalence in women who were negative for anti-CMV IgG compared to those who tested positive for anti-CMV IgG (OR: 1.44, *p* = 0.728). Similarly, in Group 2, the anti-CMV IgM seroprevalence was higher among those who tested negative for anti-CMV IgG (OR: 1.24, *p* = 0.718) (Table 5). This association was slightly stronger in Group 1 compared with Group 2.

## 4. Discussion

The study documented an ascending trend in the proportion of pregnant women under 20 years, increasing from 9.26% in 2013–2016 to 14.69% in 2019–2022. This trend aligns with other Romanian studies, which have found a higher incidence of pregnancies in young women from rural areas [12], such as 68.72% between 2003 and 2013 [24] and a threefold higher frequency in rural than urban areas between 2010 and 2014 [25]. Worldwide, according to WHO, pregnancies among teenagers have a frequency of 0.49%, and Romania is in second place in Europe in terms of teenage pregnancies with a frequency of 3.92% [24,26].

Our study identified a seroprevalence of anti-CMV IgM antibodies of 1.92% in 2013–2016 and 2.26% in 2019–2022, which is similar to seroprevalences reported in Eastern Europe by Siennicka et al. (2.2% in 2010–2011) [14] in Poland, and Vilibic-Cavlek et al. in Croatia (3.4% between 2013 and 2015) [16]. Worldwide studies have reported different seroprevalences of anti-CMV IgM antibodies in adults, ranging from 1.0% to 6.7% [27]. Additionally, in the United States, a study based on the National Health and Nutrition Examination Survey (NHANES) observed a seroprevalence of 3% in adults for anti-CMV IgM antibodies [28].

The IgM seroprevalence observed in our study is notably higher than the 0.592% reported by Neamtu et al. in 2006 for Craiova City [10]. Furthermore, in Western Romania, results of a study by Mocanu et al. on women of childbearing age found a seroprevalence of 0.342% from 2008 to 2010, which subsequently decreased to 0.291% between 2015 and 2018 [12]. These findings suggest a declining trend in the incidence of acute CMV infection in Western Romania during this period. In contrast, our study indicates an increasing trend, highlighting regional differences in the epidemiology of CMV infections.

Our investigation revealed an increase in seroprevalence in urban areas between the two studied periods (from 1.60% to 2.31%), but a slight decrease in rural areas (from 2.55% to 2.22%). Notably, in women under 20 years from Group 1, urban areas exhibited a significantly higher seroprevalence (11.11% vs. 2.86%), aligning with findings from an Iraqi study that reported a greater prevalence of acute CMV infection in urban settings (8.5%) [29]. The variation in the age demographics of women tested for CMV IgM from Group 1 to Group 2 might reflect effective public health initiatives focusing on awareness, prevention, and routine prenatal screening for CMV in pregnant women [30].

The study observed a modest increase in seroprevalence of anti-CMV IgG antibodies from 93.68% in 2013–2016 to 94.96% in 2019–2022 in South-West Romania, indicating a potentially higher immunization rate in this region.

Gorun et al. showed that in 2022, the western region of Romania had a low risk profile for primary CMV infection during pregnancy due to a high number of seropositive women. However, this risk has increased in the last ten years, from 5.4 to 8.2%, which may show the need to implement a national screening program. The study also showed a decrease in seroprevalence of anti-CMV IgG antibodies in pregnant women, from 94.6% between 2008 and 2010 to 91.8% during 2015–2018 [12], while another study from 2021 from the same region reported similar results [11].

In Europe, recent studies have reported varying seroprevalence rates, ranging from a low of 57.3% in Poland [14] to a high of 95.7% in Romania [12]. In Western Europe, the seroprevalence ranges from 45.6% in France [13] to 67.1% in Norway [15].

Our findings align with other global studies. For instance, in Latin America, anti-CMV IgG antibody seroprevalence ranged between 58.3% and 94.5%, comparable to our results, while in North America, it varied from 24.6% to 81.0% [27]. In Asia, a Korean study reported a seroprevalence of 95.8% [31], similar to our findings, whereas Japanese studies indicated lower rates between 67.2% and 70.9% [27]. African seroprevalence rates were noted at 88.5% in Ethiopia [32] and 97.5% in Sudan [33], and in Australia, the rates were around 57–58% [34,35]. These variations, particularly the lower rates in developed countries compared to developing nations, suggest that factors such as health education and socioeconomic status play significant roles in the transmission and immunization against CMV infection.

We observed in Group 1 a decrease in IgG seroprevalence from rural areas (93.97%) to urban areas (93.52%) that was maintained in Group 2 from 95.52% in rural areas to 94.27% in urban areas. Also, a declining trend from rural to urban areas was noted in Western Romania [12]. We observed a marked increase in seroprevalence in pregnant women from the 21–25 years age group between the two time periods (both in urban and rural areas). In trying to explain why the seroprevalence is high in this particular age group, it is noteworthy to mention the correlation with socioeconomic status (SES), as highlighted in one European study by Enders et al., which also showed a higher seroprevalence in young pregnant women aged 15–25 years (59.6%) [36] compared to older women, as observed in another study by the same authors that found a seroprevalence of 55.1% in pregnant women aged 21–25 years [37]. One possible explanation could be a higher proportion of women of lower socioeconomic status in this age group, as stated in a study by Friese et al. [38]. Different studies have shown that lower socioeconomic status is associated with higher rates of CMV seropositivity [37,39]. For example, a study performed in Germany reported a CMV seroprevalence of 60.1% in women with middle SES, in contrast to 46.8% in those with upper SES [39]. Other contemporary research has shown that education level can influence the CMV infection seroprevalence [15,40,41].

It is pertinent to consider that the intervals in our study (2013–2016 and 2019–2022) featured a narrower gap of three years, compared to the five-year intervals in Western Romanian studies (between 2008–2010 and 2015–2018) [12]. The proximity of our study’s second period (2019–2022) to the latter phase of Western Romanian studies (2015–2018) raises the possibility of an emerging trend in CMV infection seroprevalence.

When we compared the seroprevalence of anti-CMV IgM antibodies in immunized pregnant women versus those who were not immunized, we could not find a statistically significant difference. This lack of significant difference could suggest differences in immunization timing, individual variability of immune response, which could be influenced by genetic factors, underlying health conditions, or other concurrent infections.

### Limitations

In the analysis of the data obtained from our study, it is crucial to consider that a minority of pregnant women opted for TORCH screening in private clinics, a choice limited by the significant costs associated with this test. Therefore, the impact of this factor on the results of our study is likely minimal, as the majority of pregnant women in the South-West region of Romania prefer our center for conducting the TORCH screening, due to its financial accessibility (the tests being free of charge), thereby reducing the potential bias in interpreting the study’s data.

An additional limitation was the variance in antibody testing among participants, with some women being tested only for one type of antibody (either IgG or IgM). This led to a disparity in the number of women tested for IgM compared to those tested for IgG antibodies.

In interpreting our result, we must consider that studies show that the anti-CMV IgM antibody test in pregnant women with primary infection has a sensitivity of 55–75% and 70% for congenital infection [23].

## 5. Conclusions

In conclusion, the total IgM seroprevalence in South-West Romania has increased from 1.92% to 2.26%, and the IgG seroprevalence also increased from 93.68% to 94.96%. Regarding the area of residence, the seroprevalence of anti-CMV IgM antibodies decreased from rural areas (2.55%) to urban areas (1.60%) in the first period, while in the second period, the seroprevalence was similar in both areas. The IgG seroprevalence decreased from rural to urban areas in both time periods.

These results show a high rate of immunization against CMV in pregnant women in South-West Romania, which led to a low risk of acquiring the primary infection during pregnancy. However, the increase in the rate of primary CMV infections in pregnancy suggests the need for prioritizing screening programs and improving the existing protocols to enhance maternal and child healthcare.

## Figures and Tables

**Table 1:** Demographic characteristics of the pregnant women tested for IgM anti-CMV antibodies.

		Group 1 *n* = 572	Group 2 *n* = 2832	*p*
Area of residence (*n*/%)				
	Rural	196 (34.26%)	1618 (57.13%)	<0.001 *
	Urban	376 (65.74%)	1214 (42.87%)	
Age (years)		27.72 (5.35)	27.57 (6.30)	0.595

*: Statistically significant result.

**Table 2:** Comparative seroprevalence of anti-CMV IgM antibodies between the two groups stratified by area of residence and age group.

Variable	Group 1 2013–2016 (*n* = 572)	Group 2 2019–2022 (*n* = 2832)	*p* Value
Total prevalence	11/572 (1.92%)	64/2832 (2.26%)	0.613
Area of residence			
Rural	5/196 (2.55%)	36/1618 (2.22%)	0.769
Urban	6/376 (1.60%)	28/1214 (2.31%)	0.406
Age group			
<20 years	3/53 (5.66%)	13/416 (3.13%)	0.340
21–25 years	1/135 (0.74%)	15/700 (2.14%)	0.277
26–30 years	6/233 (2.58%)	24/777 (3.09%)	0.688
31–35 years	1/100 (1.00%)	10/615 (1.63%)	0.635
>35 years	0/51 (0.00%)	2/324 (0.62%)	0.573
Area of residence and age group			
Rural, <20 years	1/35 (2.86%)	10/318 (3.14%)	0.928
Rural, 21–25 years	1/55 (1.82%)	10/484 (2.07%)	0.901
Rural, 26–30 years	2/67 (2.99%)	12/405 (2.96%)	0.989
Rural, 31–35 years	1/28 (3.57%)	3/275 (1.09%)	0.273
Rural, >35 years	0/11 (0.00%)	1/136 (0.74%)	0.775
Urban, <20 years	2/18 (11.11%)	3/98 (3.06%)	0.122
Urban, 21–25 years	0/80 (0.00%)	5/216 (2.31%)	0.170
Urban, 26–30 years	4/166 (2.41%)	12/372 (3.23%)	0.605
Urban, 31–35 years	0/72 (0.00%)	7/340 (2.06%)	0.219
Urban, >35 years	0/40 (0.00%)	1/188 (0.53%)	0.644

**Table 3:** Demographic characteristics of female subjects tested for anti-CMV IgG antibodies.

		Group 1 *n* = 585	Group 2 *n* = 2399	*p*
Area of residence (*n*/%)				
	Rural	199 (34.02%)	1317 (54.90%)	<0.001 *
	Urban	386 (65.98%)	1082 (45.10%)	
Age (years)		27.68 (5.34)	27.86 (6.21)	0.519

*: Statistically significant result.

**Table 4:** Comparative seroprevalence of anti-CMV IgG antibodies between the two groups stratified by area of residence and age group.

Variable	Group 1 2013–2016 (*n* = 585)	Group 2 2019–2022 (*n* = 2399)	*p* Value
Total prevalence	548/585 (93.68%)	2278/2399 (94.96%)	0.215
Area of residence			
Rural	187/199 (93.97%)	1258/1317 (95.52%)	0.335
Urban	361/386 (93.52%)	1020/1082 (94.27%)	0.592
Age group			
<20 years	54/55 (98.18%)	309/328 (94.21%)	0.221
21–25 years	125/140 (89.29%)	538/563 (95.56%)	0.004 *
26–30 years	227/239 (94.98%)	641/686 (93.44%)	0.394
31–35 years	94/102 (92/.16%)	517/540 (95.74%)	0.122
>35 years	48/49 (97.96%)	273/282 (96.81%)	0.664
Area of residence and age group			
Rural, <20 years	35/36 (97.22%)	237/251 (94.42%)	0.480
Rural, 21–25 years	51/57 (89.47%)	362/377 (96.02%)	0.032 *
Rural, 26–30 years	64/67 (95.52%)	328/345 (95.07%)	0.875
Rural, 31–35 years	26/28 (92.86%)	220/230 (95.65%)	0.508
Rural, >35 years	11/11 (100.00%)	111/114 (97.37%)	0.586
Urban, <20 years	19/19 (100.00%)	72/77 (93.51%)	0.254
Urban, 21–25 years	74/83 (89.16%)	176/186 (94.62%)	0.106
Urban, 26–30 years	163/172 (94.77%)	313/341 (91.79%)	0.218
Urban, 31–35 years	68/74 (91.89%)	297/310 (95.81%)	0.162
Urban, >35 years	37/38 (97.37%)	162/168 (96.43%)	0.772

*: Statistically significant result.

**Table 5:** Prevalence of IgM and anti-CMV IgG antibodies.

	Group 1 (2013–2016)			Group 2 (2019–2022)		
	Anti-CMV IgG (−)	Anti-CMV IgG (+)	Total	Anti-CMV IgG (−)	Anti-CMV IgG (+)	Total
Anti-CMV IgM (+)	1 (2.70%)	10 (1.89%)	11 (1.94%)	3 (2.52%)	46 (2.08%)	49 (2.10%)
Anti-CMV IgM (−)	36 (97.30%)	520 (98.11%)	556 (98.06%)	116 (97.48%)	2164 (97.92%)	2280 (97.90%)
Total	37 (100%)	530 (100%)	567 (100%)	119 (100%)	2210 (100%)	2329
Odds	0.028	0.019	0.020	0.026	0.021	0.021
Odds Ratio	1.44			1.24		
*p* value	0.728			0.718		
95% CI	0.032–10.666			0.244–3.957		

## Data Availability

All data are available upon request from the corresponding author.
